# Optimization of Mucosal Responses after Intramuscular Immunization with Integrase Defective Lentiviral Vector

**DOI:** 10.1371/journal.pone.0107377

**Published:** 2014-09-11

**Authors:** Alessandra Rossi, Zuleika Michelini, Pasqualina Leone, Martina Borghi, Maria Blasi, Roberta Bona, Massimo Spada, Felicia Grasso, Alessio Gugliotta, Mary E. Klotman, Andrea Cara, Donatella Negri

**Affiliations:** 1 Department of Infectious, Parasitic and Immune-mediated Diseases, Istituto Superiore di Sanità, Rome, Italy; 2 Department of Therapeutic Research and Medicine Evaluation, Istituto Superiore di Sanità, Rome, Italy; 3 Department of Cell Biology and Neuroscience, Istituto Superiore di Sanità, Rome, Italy; 4 Department of Hematology, Oncology and Molecular Medicine, Istituto Superiore di Sanità, Rome, Italy; 5 Department of Medicine, Duke University Medical Center, Durham, North Carolina, United States of America; Centers for Disease Control and Prevention, United States of America

## Abstract

Many infectious agents infiltrate the host at the mucosal surfaces and then spread systemically. This implies that an ideal vaccine should induce protective immune responses both at systemic and mucosal sites to counteract invasive mucosal pathogens. We evaluated the *in vivo* systemic and mucosal antigen-specific immune response induced in mice by intramuscular administration of an integrase defective lentiviral vector (IDLV) carrying the ovalbumin (OVA) transgene as a model antigen (IDLV-OVA), either alone or in combination with sublingual adjuvanted OVA protein. Mice immunized intramuscularly with OVA and adjuvant were compared with IDLV-OVA immunization. Mice sublingually immunized only with OVA and adjuvant were used as a positive control of mucosal responses. A single intramuscular dose of IDLV-OVA induced functional antigen-specific CD8+ T cell responses in spleen, draining and distal lymph nodes and, importantly, in the *lamina propria* of the large intestine. These results were similar to those obtained in a prime-boost regimen including one IDLV immunization and two mucosal boosts with adjuvanted OVA or vice versa. Remarkably, only in groups vaccinated with IDLV-OVA, either alone or in prime-boost regimens, the mucosal CD8+ T cell response persisted up to several months from immunization. Importantly, following IDLV-OVA immunization, the mucosal boost with protein greatly increased the plasma IgG response and induced mucosal antigen-specific IgA in saliva and vaginal washes. Overall, intramuscular administration of IDLV followed by protein boosts using the sublingual route induced strong, persistent and complementary systemic and mucosal immune responses, and represents an appealing prime-boost strategy for immunization including IDLV as a delivery system.

## Introduction

Many infections start at mucosal surfaces and then spread throughout the body. Therefore an ideal vaccine should induce protective immune responses both at mucosal sites, such as respiratory, gastrointestinal and genitourinary tracts, and at a systemic level. Mucosal immune responses are usually achieved by delivering vaccine formulations through oral, intranasal and vaginal routes [1] and with the use of appropriate adjuvants that can induce systemic immune responses as well [2]. The sublingual mucosa has recently emerged as an attractive alternative mucosal immunization route in preclinical models [3]. Sublingual administration of different vaccine formulations elicits strong antigen-specific immune responses in different mucosal sites and at the systemic level [4]–[9]. In some cases the sublingual route has proved to be safer than the intranasal route for vaccine delivery [4], [10], [11]. However, a strong mucosal adjuvant and/or an appropriate delivery system are needed to elicit a strong immune response after sublingual immunization, especially in large animal models and in humans [2], [12]. Vaccine potency can be improved through a mixed-modality strategy, including heterologous vaccination, based on the use of recombinant vectors and soluble antigens [13]. Indeed, in a recent clinical trial, an immunization regimen combining priming with a recombinant canarypox vector vaccine plus two booster injections of recombinant HIV-1 gp120 protein, significantly reduced the cases of HIV infection in a risk population, with a trend towards prevention [14]. Several reports have suggested that combinations of mucosal and systemic immunizations may enhance both mucosal and systemic immune responses [15]–[19]. To this regard, and to further amplify the potency of a vaccine also in terms of mucosal responses, a heterologous prime-boost schedule of immunization could be helpful in inducing a comprehensive immune response in terms of antigen-specific antibodies and T cells at mucosal and systemic levels.

Integrase defective lentiviral vectors (IDLVs) represent a powerful tool to deliver foreign genes. IDLVs are safer than their integrase competent counterparts, since they lack integrase activity, and transgene expression is efficiently driven from unintegrated circular forms of the vector genome [20]–[22]. Several reports have shown that IDLVs are suitable for delivery of vaccine antigens in preventive vaccine strategies [23]–[29], demonstrating that immunization with IDLVs induced strong and protective antigen-specific immune responses, in absence of vector integration. Moreover, we recently demonstrated that therapeutic vaccination with IDLV expressing HPV-E7 as a tumor antigen results in eradication of TC-1 derived tumor in tumor-bearing mice [30]. However, while the efficacy of intramuscular immunization with IDLV at inducing systemic antigen-specific immune responses after a single immunization is well established, no data are available concerning the mucosal immune responses. Some reports showed that in addition to systemic T cell response, intramuscular immunization was able to induce CD8+ T cell mediated antigen-specific immunity in gut mucosa, an important portal of entry of many infectious pathogens [31], [32].

In the present study, we analyzed the antigen-specific immune response using different immunization protocols, by focusing on mucosal cellular and antibody responses induced by a single intramuscular administration of IDLV expressing ovalbumin protein (OVA) as a model antigen either alone or in combination with sublingual adjuvanted OVA in prime-boost regimens. Results indicate that intramuscular immunization with IDLV is sufficient to induce a persistent CD8+ T cell response in the *lamina propria* of large intestine, while a mucosal protein boost is necessary for the induction of mucosal IgA.

## Materials and Methods

### Vector construction and production

Transfer vector plasmid pTY2CMV-GFPW expressing GFP has been already described [33]. For construction of transfer vector expressing OVA protein, the coding sequence for OVA was excised from plasmid pOVA, kindly provided by Dr Piergiuseppe de Berardinis (I.B.P., C.N.R., Naples, Italy), using SnaBI/XbaI and cloned into the transfer vector pTY2CMV-GFPW by replacing the GFP coding sequence, thus obtaining the transfer vector plasmid pLenti-OVA. The HIV-based packaging plasmid IN defective (pcHelp/IN-) and the pseudotyping VSV.G envelope-expressing (pMD.G) plasmids have been already described [23], [34], [35].

For production of recombinant IDLV expressing OVA (IDLV-OVA), 293T cells were transiently transfected on 10 cm Petri dishes using the Calcium Phosphate-based Profection Mammalian Transfection System (Promega Corporation, Madison WI, USA) as previously described [23]. For concentration, vector containing supernatants were ultracentrifuged (Beckman Coulter, Inc., Fullerton, CA, USA) on a 20% sucrose gradient (Sigma Chemical Co. St. Louis, MO, USA) and viral pellets were resuspended in 1× PBS. Viral titers for IDLV-OVA were performed by the reverse transcriptase (RT) activity assay [36] over standards of known infectivity and the vector-associated RT activity were compared with the ones of IDLV-GFP virions of known infectious titers and RT activity, thus allowing for the determination of their infectious titer units [37].

### Western blot, DNA isolation and PCR

To verify the expression of ovalbumin (OVA), 293T cells were seeded in 10 cm plates and transduced with IDLV-OVA or IDLV-GFP at 37°C in atmosphere containing 5% CO2. Thirty-six hours post-transduction supernatants and cells were collected. Equivalent amounts of cells transduced with IDLV-OVA or IDLV-GFP were lysed in lysis buffer (20 mM HEPES, 50 mM NaCl, 10 mM EDTA, 2 mM EGTA, 0.5%, NP-40, 50 mM NaF, 1 mM orthovanadate, 1 mM PMSF, 5 µg/ml of aprotin and 5 µg/ml of leupeptin). Proteins of cell lysates and supernatants were separated on 12% SDS polyacrylamide gel along with the purified OVA protein (Sigma), used as a positive control and transferred to a nitrocellulose membrane (GE HEALTHCARE). The filters were saturated overnight with 5% non fat dry milk (NFDM) in PBST (PBS with 0.1% Tween 20) and then incubated with a rabbit anti-OVA polyclonal antibody (AB1225, Millipore) for 1 hr at room temperature, followed by incubation for 1 hr at room temperature with an anti-rabbit HRP-conjugated IgG (Sigma). The immunocomplexes were visualized using chemiluminescence ECL detection system (Luminata Crescendo Western HRP Substrate, Millipore) [30].

DNA from muscle, splenocytes and large intestine was extracted using the SV Total RNA Isolation System protocol, modified for DNA preparation (Promega Corporation, Madison, WI) [38]. All samples supported the amplification of the mouse glyceraldehyde 3-phosphate dehydrogenase gene (G3PDH), (GlymoFor: 5'-TGAAGGTCGGTGTGAACGGATTTGGC-3'; GlymoRev: 5'-CATGTAGGCCATGAGGTCCACCAC-3') and were included in subsequent PCR analysis to detect the presence of the vector DNA sequence using 500 ng of DNA and a primer pair spanning the LTR region at the 3′ end of the vector (PPTs: 5′-CAGCTGTAGATCTTAGCCACT-3′; AA55: 5′-CTGC TAGAGATTTTCCACACTGAC-3′), as described [23]. PCR parameters were: 1 cycle of 5 min at 94°C, followed by 40 cycles of 30 sec at 94°C, 30 sec at 60°C, 30 sec at 72°C with a final extension step of 10 min at 72°C in a 9700 Perkin-Elmer Thermocycler.

### Mice and immunization schedule

#### Ethics statement

Animals were maintained under specific pathogen-free conditions in the animal facilities at the Istituto Superiore di Sanità and treated according to European Union guidelines and Italian legislation (Decreto Legislativo 116/92, implementing the 86/609/CEE Directive on laboratory animal protection). All animal studies were reviewed and approved by the Service for Biotechnology and Animal Welfare at the Istituto Superiore di Sanità (ISS registration n. 3138 of 26/01/2012). All animals were euthanized by CO_2_ inhalation using approved chambers, and efforts were made to minimize suffering and discomfort.

C57/Bl6 female mice were purchased from Charles River Laboratories, Calco, Italy. A scheme of immunization protocols is described in Table 1. Mice were immunized by intramuscular (i.m.) injection with IDLV expressing OVA (IDLV-OVA) either alone (group A) or in combination with two doses of OVA protein (OVAp) plus E.coli heat-labile enterotoxin adjuvant (LT), delivered sublingually either after or before IDLV-OVA (group B and C, respectively). Other groups included mice immunized once intramuscularly with OVAp plus LT alone (group D) or in combination with two sublingual (s.l.) doses of OVAp + LT (groups E). A group of mice representing the positive control for mucosal immune responses was sublingually immunized with 4 doses of OVAp + LT (group F); the number of doses was selected based on our previous data [6] and in order to obtain a strong positive control for mucosal humoral and cellular responses. Naïve, non-immunized mice were kept for parallel analysis. All immunizations were given 2 weeks apart. IDLV-OVA (1.1×10^7^ RT units total/mouse) was administered in both left and right thigh. The same dose of OVAp (20 µg/mouse per dose) + LT (1 µg/mouse per dose) was injected either intramuscularly in one thigh or sublingually. Sublingually immunized mice (5 µl/mouse per dose) were deeply anesthetized with ketamine (2 mg/mouse) and xylazine (0.17 mg/mouse), in order to avoid the swallowing of saliva during the immunization, as already described [6].

**Table 1:** Vaccine regimens.

Group	1^st^ immunization week 0	2^nd^ immunization week 2	3^rd^ immunization week 4	4^th^ immunization week 6
**A**	i.m. IDLV-OVA	-	-	-
**B**	i.m. IDLV-OVA	s.l. OVAp + LT	s.l OVAp + LT	-
**C**	s.l. OVAp + LT	s.l. OVAp + LT	i.m. IDLV-OVA	-
**D**	i.m. OVAp + LT	-	-	-
**E**	i.m. OVAp + LT	s.l. OVAp + LT	s.l. OVAp + LT	-
**F**	s.l. OVAp + LT	s.l. OVAp + LT	s.l. OVAp + LT	s.l. OVAp + LT
**Naive**	-	-	-	-

i.m.: intramuscular; s.l.: sublingual; OVAp: ovalbumin protein; LT: E.coli heat-labile enterotoxin.

The cellular immune responses were analyzed at 2 weeks and at 6 months after the last immunization, by sacrificing 4 mice per group at each time point and each experiment was repeated at least two times. Anti-OVA IgG and IgA antibodies (Abs) were measured in plasma and mucosal secretions at 2 weeks after each immunization and at 6 months after the last immunization.

Plasma samples were obtained from blood collected from the retro-orbital plexus of mice with heparin-treated glass Pasteur pipettes and stored at −20°C until assayed. Saliva was collected after intraperitoneal injection of pilocarpine (160 µg/mouse). Vaginal washes were obtained introducing 50 µl of PBS each for three times into the vaginal tract of mice using a Gilson pipette. At the time of sacrifice, spleen, lymph nodes (submandibular, mesenteric, inguinal) and large intestine were recovered for the analysis of cellular immune responses.

### Preparation of single-cell suspensions

Splenocytes and lymph node derived cells were prepared by mechanical disruption and passage through cell strainers (BD Pharmingen, San Diego, CA, USA) and resuspended in RPMI 1640 (Euroclone) containing 10% fetal bovine serum (FBS) (Lonza), 100 units/ml of penicillin–streptomycin–glutamine (Euroclone), non-essential aminoacids (Euroclone), sodium pyruvate 1 mM (Euroclone), HEPES buffer solution 25 mM (Euroclone) 50 µM 2-mercaptoethanol (Sigma Chemicals). In order to isolate *lamina propria* (LP) lymphocytes, the large intestine surgically removed from sacrificed mice was cleaned, cut longitudinally and then sliced into small pieces with a scalpel. Tissue fragments were incubated shaking with 15 ml of Hank's balanced salt solution (HBSS) (Euroclone), 10% FBS, Hepes buffer solution 25 mM (Euroclone), EDTA 5 mM (Sigma Chemicals) and dithiothreitol (DTT) 1 mM (Sigma Chemicals) for 15 minutes at 37°C. Supernatants were discarded and left fragments were spun down, resuspended in liberase (800 units/sample, Roche) and DNAse I (40 units/sample, Roche, Monza, Italy) and incubated shaking for 1 hour at 37°C. After incubation both supernatants and pellets were filtered through 100 µm cell strainers, resuspended in 20 ml Percoll 30%-EDTA 1 mM (Sigma Chemicals) and centrifuged at 290xg for 25 minutes. LP lymphocytes were recovered in the pellet. Cells obtained from the same immunization group were pooled to ensure there was a sufficient number for subsequent tests.

### IFNγ ELISPOT and dextramer staining

The IFNγ ELISPOT assay was performed using the BD ELISPOT kit reagents and protocol (BD Biosciences). Briefly, single cell suspensions from spleen and lymph nodes were seeded at a density of 2×10^5^/well in 96 well plates and stimulated overnight either with 2 µg/ml of the H-2K^b^ restricted OVA 8mer peptide (SIINFEKL) or with 5 µg/ml of concanavalin A (Sigma Chemicals) used as a positive control. Complete medium treated cells were used as negative controls. Spot Forming Cells (SFC) were counted with an ELISPOT reader (A.EL.VIS, Hannover, Germany) and results expressed as IFNγ secreting cells/10^6^ cells.

For dextramer staining, cells from lymph nodes and large intestine *lamina propria* were washed once with 2 ml of PBS-5% bovine serum albumin (BSA) (Sigma Chemicals) in 5 ml polystyrene tubes and centrifuged. After discarding the supernatant, 1×10^6^ cells were resuspended in residual volume (50 µl) and 10 µl of H-2K^b^-SIINFEKL R-PE conjugated dextramer (Immudex, Copenhagen, Denmark) was added in each sample for 10 minutes at room temperature in the dark. Cells were washed again and PerCP-Cy5.5 conjugated anti-mouse CD8a (BD Pharmigen) and anti-mouse CD3 FITC (Immunological Sciences, Rome, Italy) were added for 20 minutes on ice in the dark. Cells were washed twice, resuspended in 0.5 ml PBS-1% paraformaldeyde and analyzed at the FACScalibur (BD Biosciences).

### Intracellular staining for cytokines

Splenocytes were either cultured in the presence of OVA-specific 8mer peptide (5 µg/ml) or left untreated in the presence of anti-mouse CD28 mAb (BD Pharmigen) at 2 µg/ml. PMA (10 ng/ml) (Sigma Chemicals) in combination with Ionomicin (1 µg/ml) (Sigma Chemicals) were used as positive control. One hour after stimulation, 10 µg/ml of Brefeldin A (Sigma Chemicals) was added to the culture to inhibit cytokine secretion and cells were incubated overnight at 37°C. After blocking of Fc receptors by treatment with anti-mouse CD16/CD32 (BD Pharmigen) cells were stained with fluorochrome conjugates FITC anti-mouse CD3 (Immunological Sciences), PE anti-mouse CD4 (Immunological Sciences) and PerCP Cy 5.5 anti-mouse CD8a (BD Pharmigen). Cells were washed, fixed with 4% paraformaldehyde (Sigma Chemicals), permeabilized in PBS-0.5% saponin (Sigma Chemicals) and stained with APC conjugated anti-mouse IFNγ and PE-Cy 7 conjugated anti-mouse TNFα or their isotype-matched controls (BD Pharmigen). Samples were washed and analyzed by FACScanto (BD Biosciences)

### Measurement of OVA-specific IgG and IgA antibodies

Plasma, saliva and vaginal washes were tested for the presence of anti-OVA IgG or IgA antibodies by a standard ELISA. Ninety-six well plates (Greiner bio-one, Germany) were coated with 0.5 µg/well of OVA overnight at 4°C. After washing and blocking for 2 hrs with 200 µl of PBS containing 1% BSA (Sigma Chemicals), serial dilutions of plasma and mucosal secretions from individual mice were added to wells in duplicate and incubated for 2 hrs at room temperature. The plates were washed and biotin-conjugated goat anti-mouse IgG (Southern Biotech, Birmingham, AL, USA) or IgA (Southern Biotech) was added to the wells for 2 hrs at room temperature. The plates were washed again before the addition of horse radish peroxidase (HRP)-conjugated streptavidin (AnaSpec, Fremont, CA, USA) for 30 min at room temperature. The antigen–antibody reaction was measured by using the 3.3,5.5-tetramethylbenzidine substrate (SurModics BioFX, Edina, MN, USA) and the reaction was stopped with 50 µl of H_2_SO_4_ 1M. Endpoint titers were determined as the reciprocal of the highest dilution giving an absorbance value at least equal to threefold that of background (biological sample from naïve mice). For each group of immunization, results were expressed as mean titer ± standard deviation.

### Statistical analysis

The immune responses were expressed as averages ± standard deviation. Statistical significance was determined by unpaired two tailed t-Student test. Paired two tailed t-Student test was used when appropriate and specified in the text; p<0.05 was considered statistically significant.

## Results

### Ovalbumin is efficiently expressed from IDLV

**Figure 1:**
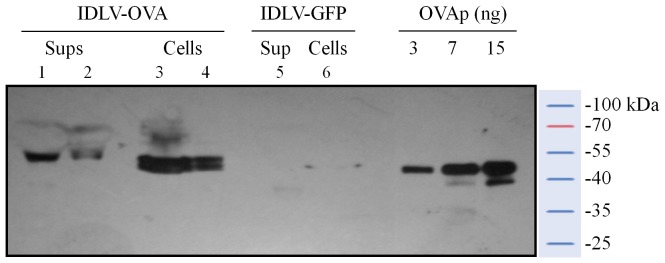
Expression of ovalbumin from IDLV. Western blot analysis of cell lysates and supernatants from 293T cells transduced with IDLV-OVA or IDLV-GFP as negative control, using an anti-OVA polyclonal antibody. Ovalbumin protein (OVAp, predicted size 47 kD) was used as positive control. Samples included supernatants showing secreted OVA (lane 1 and lane 2, 14 µl and 7 µl from 293T cells cultured at 5×10^5^/ml, respectively), and cell lysates (lane 3 and lane 4, corresponding to 3×10^5^ and 0.75×10^5^ cells equivalent, respectively). Supernatants (lane 5, 14 µl) and cell lysates (lane 6, 3×10^5^ cells equivalent) from IDLV-GFP transduced cells did not produce OVA. OVA protein (OVAp) at indicated amounts was used as positive control.

To confirm expression of ovalbumin (OVA) from IDLV-OVA, 293T cells were transduced with IDLV-OVA or IDLV-GFP as a control and cell lysates were analyzed by Western blotting assay. As shown in Figure 1, a band corresponding to the full length OVA was detected in cells transduced with IDLV-OVA but not in cells transduced with IDLV-GFP. Importantly, 293T cells transduced with IDLV-OVA released OVA protein in the supernatant. These results demonstrated that OVA protein was efficiently expressed *in vitro* from IDLV, validating IDLV-OVA as a suitable candidate for in vivo vaccination studies.

### Intramuscular administration of IDLV-OVA either alone or in prime-boost regimens induces strong systemic antigen-specific T cell responses

**Figure 2:**
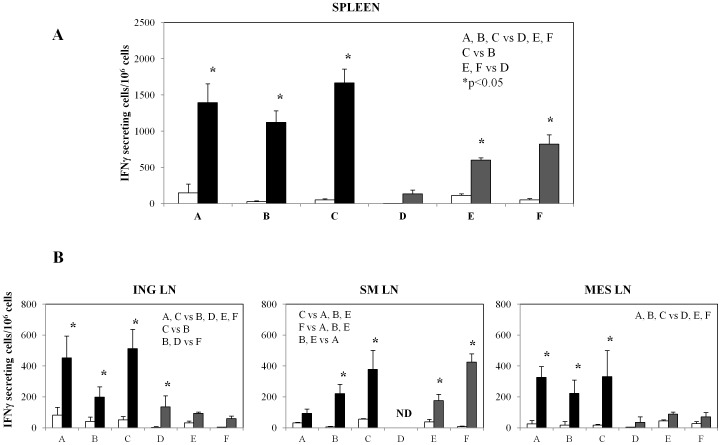
Analysis of systemic antigen-specific CD8+ T cell response at the peak of immune response. Two weeks after the final immunization, animals from all groups (vaccination regimens are described in Table 1) were sacrificed and cells from different sites were used to perform INFγ ELISPOT. Splenocytes (**A**) and lymphocytes from draining and distal lymph nodes (**B**) were stimulated overnight with medium alone (blank bars) or with H-2K^b^ restricted OVA-specific 8mer peptide (SIINKFEL) (filled bars). Results are expressed as mean IFNγ secreting cells (measured as spot forming cells)/10^6^ cells presented as group means ± standard deviations. ING, SM and MES LN: inguinal, submandibular and mesenteric lymph nodes, respectively. The asterisks specify statistically significant differences (p<0.05) between groups indicated within the graph.

Groups of mice were immunized according to the schedule of immunization shown in Table 1. Two weeks after the final immunization mice from all groups were sacrificed, lymphoid organs were removed and the presence of antigen-specific T cells was evaluated by IFNγ ELISPOT assay. Splenocytes derived from all immunized groups showed IFNγ secreting cells upon stimulation with the H-2K^b^ restricted OVA 8mer peptide (Figure 2A). In particular, mice immunized with IDLV-OVA either alone, as a prime or as a boost (groups A, B and C, respectively) showed high numbers of antigen-specific IFNγ-producing cells (1395±150, 1122±158 and 1665±191 SFC/10^6^cells, groups A, B and C, respectively). Of note, two s.l. boosts with OVAp + LT (group B) did not increase the number of IFNγ-producing T cells compared to group A, while IDLV boost in group C induced the highest number of IFNγ secreting cells compared to groups A and B (p<0.05 C vs B; p>0.05 A vs C). The lowest response was detected in animals intramuscularly immunized once with OVAp + LT (group D, 136±51 SFC/10^6^ cells). This response increased when animals were sublingually boosted twice with the protein (group E, 600±34 SFC/10^6^ cells), without reaching the levels observed in IDLV-immunized animals.

In mice sublingually immunized four times (group F) the cellular immune response was higher (823±126 SFC/10^6^cells) than that observed in group D (p<0.05) and group E, but significantly lower than that present in the IDLV-immunized groups (p<0.05). Splenocytes derived from untreated naïve mice did not show any specific IFNγ response (data not shown).

Lymph node-derived cells from mice of the same groups were also utilized for the analyses, and the results are summarized in Figure 2B. In inguinal lymph nodes (ING LN), draining the thigh muscle region (injection site), groups A and C showed a similar number of antigen-specific IFNγ producing T cells (425±82 and 512±51 SFC/10^6^cells, respectively), which was significantly higher than that present in mice from all other groups (198±42, 136±3, 93±35 and 60±4 SFC/10^6^cells, B, D, E and F groups, respectively). In submandibular lymph nodes (SM LN), draining the sublingual site, mice receiving OVA sublingually showed a higher response (222±6, 378±56, 170±38 and 424±8 SFC/10^6^cells, B, C, E and F groups, respectively) compared to the animals immunized with IDLV-OVA alone (93±32 SFC/10^6^cells). IFNγ producing T cells were also detected in mesenteric lymph nodes (MES LN), which represents a distal site from immunizations. In this case all the IDLV-immunized groups (327±25, 222±17 and 331±17 SFC/10^6^cells, A, B and C, respectively) showed a significantly higher response compared to the other groups (34±4, 89±45 and 70±27 SFC/10^6^cells, D, E and F, respectively). Lymph node derived cells from untreated naïve mice did not show any specific IFNγ response (data not shown).

These results indicate that all immunized mice have a systemic antigen-specific T cell response and that mice immunized intramuscularly with IDLV-OVA showed overall higher responses compared to the animals immunized with the adjuvanted OVAp, administered either i.m. or s.l.

### A single intramuscular injection with IDLV-OVA induces antigen-specific CD8+ T cell response in gut mucosa

**Figure 3:**
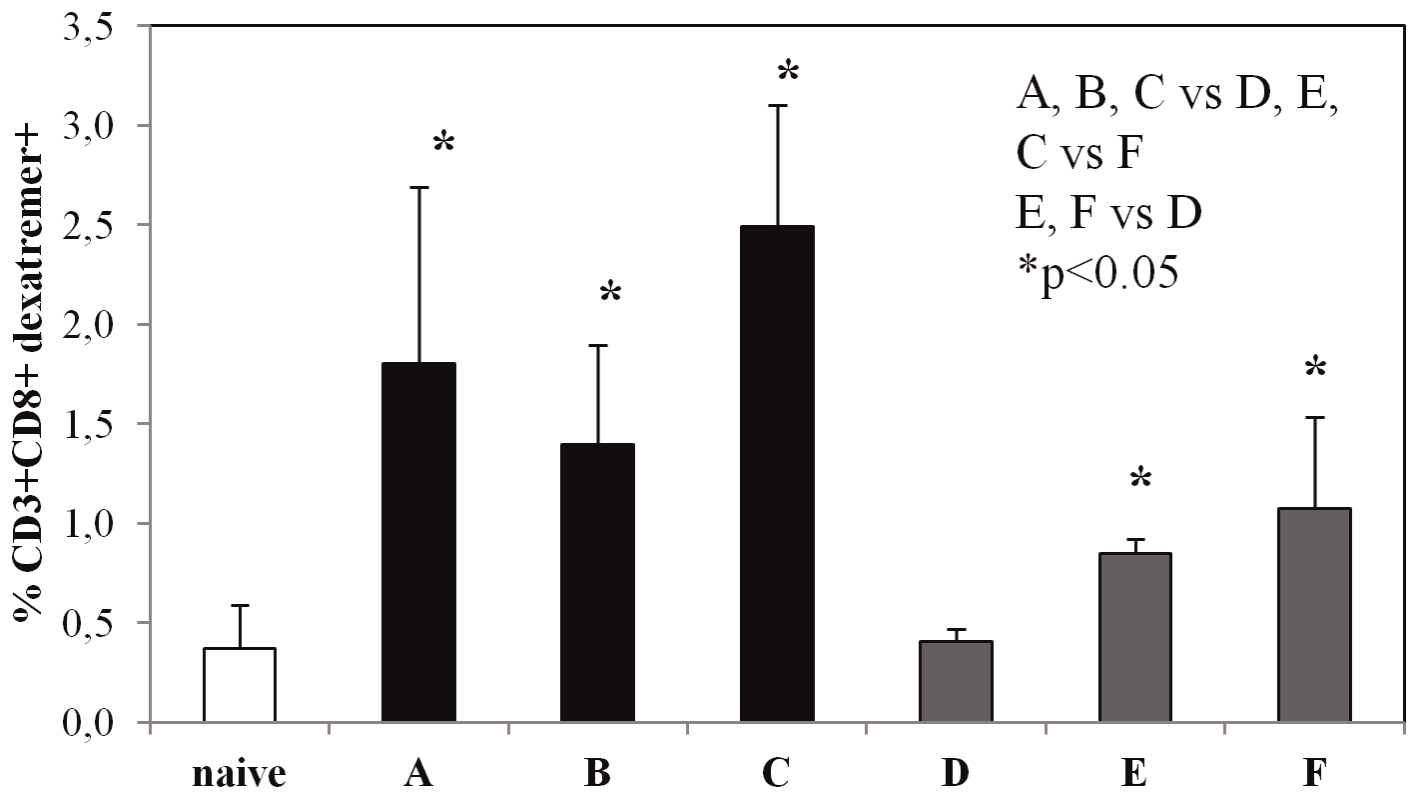
Frequency of mucosal antigen-specific CD8 T cells. Two weeks after the final immunization, mice from all groups (vaccination regimens are described in Table 1) were sacrificed. Lymphocytes derived from large intestine *lamina propria* were stained with fluorescent H-2K^b^-SIINKFEL dextramers, anti-mouse CD3 and anti-mouse CD8 and analyzed by FACScalibur. The analysis was performed on gated CD3+CD8+ cells from immunized or naïve mice. Results are expressed as percentage of CD3+CD8+ dextramers+ cells presented as group means ± standard deviations.

The presence of antigen-specific CD8+ T cells in the *lamina propria* (LP) of large intestine was evaluated by staining LP derived lymphocytes with H-2K^b^-SIINFEKL dextramers, binding to TCR specific for the OVA MHC class I-restricted epitope. Results are shown in Figure 3, indicating the percentage of CD3+CD8+dextramer+ cells from immunized groups and naive animals, used as negative control for background of dextramer staining. I.m. immunization with IDLV-OVA alone or in prime-boost regimens (A, B and C groups), but not with adjuvanted OVAp (group D), induced an antigen-specific CD8+ T cell immune response in the gut at 2 weeks after the last immunization. Of note a single i.m. immunization with IDLV, but not with OVAp + LT, was sufficient to induce CD8+T cells in the mucosa. As expected, a detectable response was observed also in mice mucosally immunized with OVAp + LT (group E and F).

### Sublingual immunization with protein is necessary for induction of mucosal IgA antibodies

To evaluate antigen-specific antibodies at systemic and mucosal sites, OVA-specific IgG were measured in plasma at 2 weeks after each immunization while IgA were measured in saliva and vaginal washes (VW) at 2 weeks after the final immunization.

**Figure 4:**
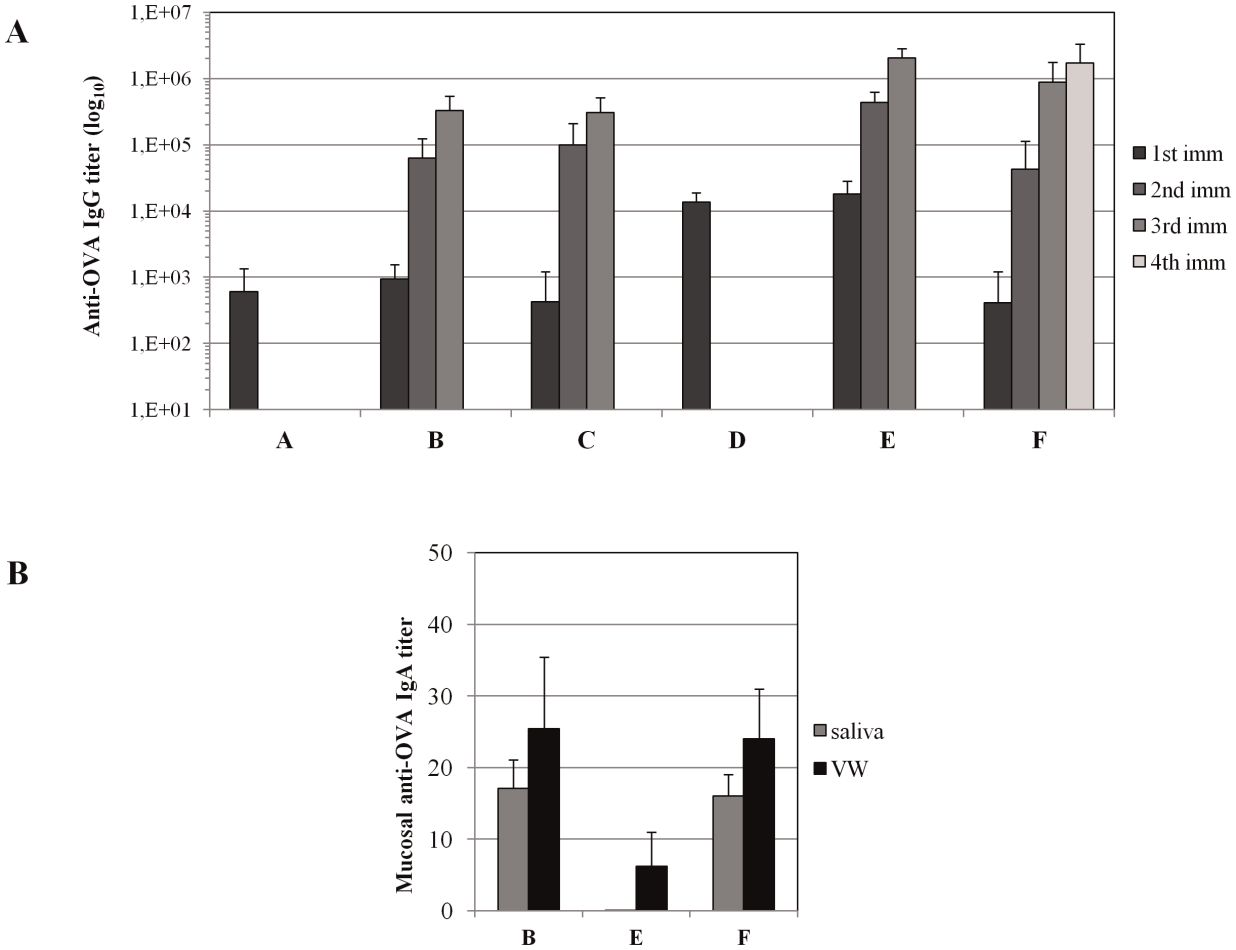
Analysis of antigen-specific antibodies in plasma and mucosal secretions. (**A**) Kinetics of plasma anti-OVA IgG titers in mice belonging to different groups (vaccination regimens are described in Table 1) at 2 weeks after each immunization. Results are expressed as mean titer presented as group means ± standard deviations. The statistical analysis is described and discussed in the text. (**B**) Analysis of anti-OVA IgA titer in saliva and vaginal washes (VWs) collected 2 weeks after the final immunization. Results are expressed as mean titer presented as group means ± standard deviations.

Figure 4A shows that i.m. immunization with IDLV-OVA alone induced low but detectable anti-OVA IgG in plasma (group A and B after 1^st^ immunization) that significantly increased after each boost (group B). Comparable levels of IgG were observed in plasma of mice immunized twice mucosally with OVAp + LT and boosted intramuscularly with IDLV-OVA (Group C). A single i.m. immunization with adjuvanted OVAp (group D) induced a significantly higher level of IgG compared to group A. The titer further increased after two s.l. boosts (group E). Finally, in group F, a single s.l. immunization induced anti-OVA IgG levels comparable to those obtained after a single i.m. IDLV-OVA immunization (group A and B) but significantly lower than those observed in group D and E. Overall, i.m. priming and s.l. boosts with OVAp + LT (group E) induced the highest levels of IgG in plasma after each immunization, when compared to the other groups. In all cases, within the same group, IgG titers significantly increased after each boost (p<0.05, paired two tailed t-Student test was used).

IgA measurement in mucosal secretions showed a more composite picture (Fig. 4B). In accordance with our previous study [6], multiple s.l. immunizations (group F) induced antigen-specific IgA antibodies both in saliva and vaginal washes. I.m. immunization alone either with IDLV or with protein and adjuvant did not induce anti-OVA IgA antibodies (group A and D, data not shown). IgA were induced after two sublingual boosts with adjuvanted OVA in both mucosal secretions from group B mice, while only in vaginal washes and at a lower level from group E animals. Interestingly, while anti-OVA IgA were present in group B mice, receiving i.m. IDLV-OVA followed by two s.l. boost with OVAp + LT, they were absent in group C mice (data not shown), receiving two s.l. adiminstration of OVAp + LT followed by i.m. IDLV-OVA. This was unexpected, since both groups received IDLV-OVA and OVAp + LT, delivered sublingually, although using different prime-boost regimens.

### Intramuscular injection with IDLV-OVA induces long lasting systemic and mucosal antigen-specific CD8+ T cell response

**Figure 5:**
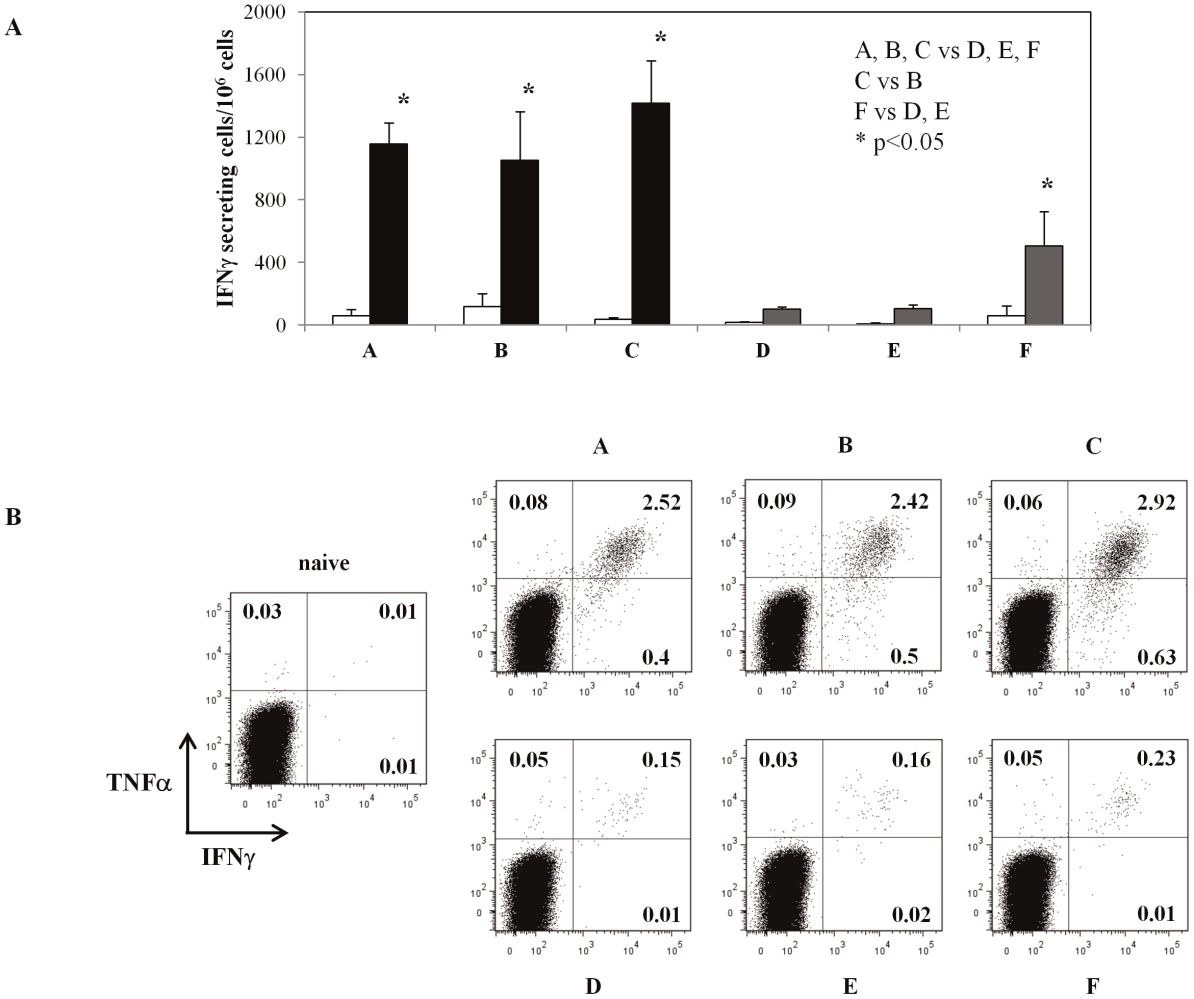
Persistence of systemic antigen-specific CD8+ T cell response. Six months after the last immunization mice from all groups (vaccination regimens are described in Table 1) were sacrificed and splenocytes used for the analysis of OVA-specific T cells. (**A**) IFNγ ELISPOT. Splenocytes were stimulated overnight with medium alone (blank bars) or with H-2K^b^ restricted OVA-specific 8mer peptide (SIINFEKL) (filled bars). IFNγ-producing T cells are expressed as the number of spot forming cells per 10^6^ cells. Results are presented as group means ± standard deviations. The asterisks indicate statistically significant differences (p<0.05) between indicated groups. (**B**) Analysis of multifunctional antigen-specific CD8+ T lymphocytes by intracellular assay for IFNγ and TNFα production. A representative experiment is shown. The analysis was performed on gated CD3+CD8+ cells from immunized or naïve mice. The percentages of single or double-cytokine producing cells were calculated and are indicated within the dot plots.

Persistence of antigen-specific cell-mediated immunity was analyzed in all groups of mice at 6 months after the final immunization by IFNγ ELISPOT assay, intracellular staining (ICS) on IFNγ and TNFα producing CD8+ T cells, and detection of antigen-specific CD8+ T cells by dextramer staining. The IFNγ ELISPOT assay showed that IFNγ producing OVA-specific cells were still detectable in splenocytes from all groups of mice (Fig. 5A). In particular, mice receiving IDLV-OVA (groups A, B and C) showed the highest number of IFNγ producing cells in spleens (averages of 1158±133, 1052±311 and 1416±272 SFC/10^6^ splenocytes, respectively; C vs B p<0.05), which were significantly higher than the values observed in groups D, E and F (group D 101±16 SFC/10^6^, group E 103±26 SFC/10^6^, group F, 506±219 SFC/10^6^; p<0.05). This was in line with the results obtained at 2 weeks from the last immunization (Fig. 2A). To verify the functionality of OVA-specific CD8+ T cells at several months from the last immunization, multifunctional OVA-specific CD8+ T cell responses were analyzed by ICS for IFNγ and TNFα. A representative experiment is shown in Figure 5B. CD8+ T cells producing both cytokines were evident in all groups upon stimulation with the specific OVA peptide, confirming the ELISPOT data. Groups A, B and C showed remarkable percentages of multifunctional CD8+ T cells, which were significantly higher than those present in all other groups. We did not find evidence of CD8 negative T cells producing either IFNγ or TNFα upon stimulation with the H-2K^b^ specific OVA peptide (data not shown).

**Figure 6:**
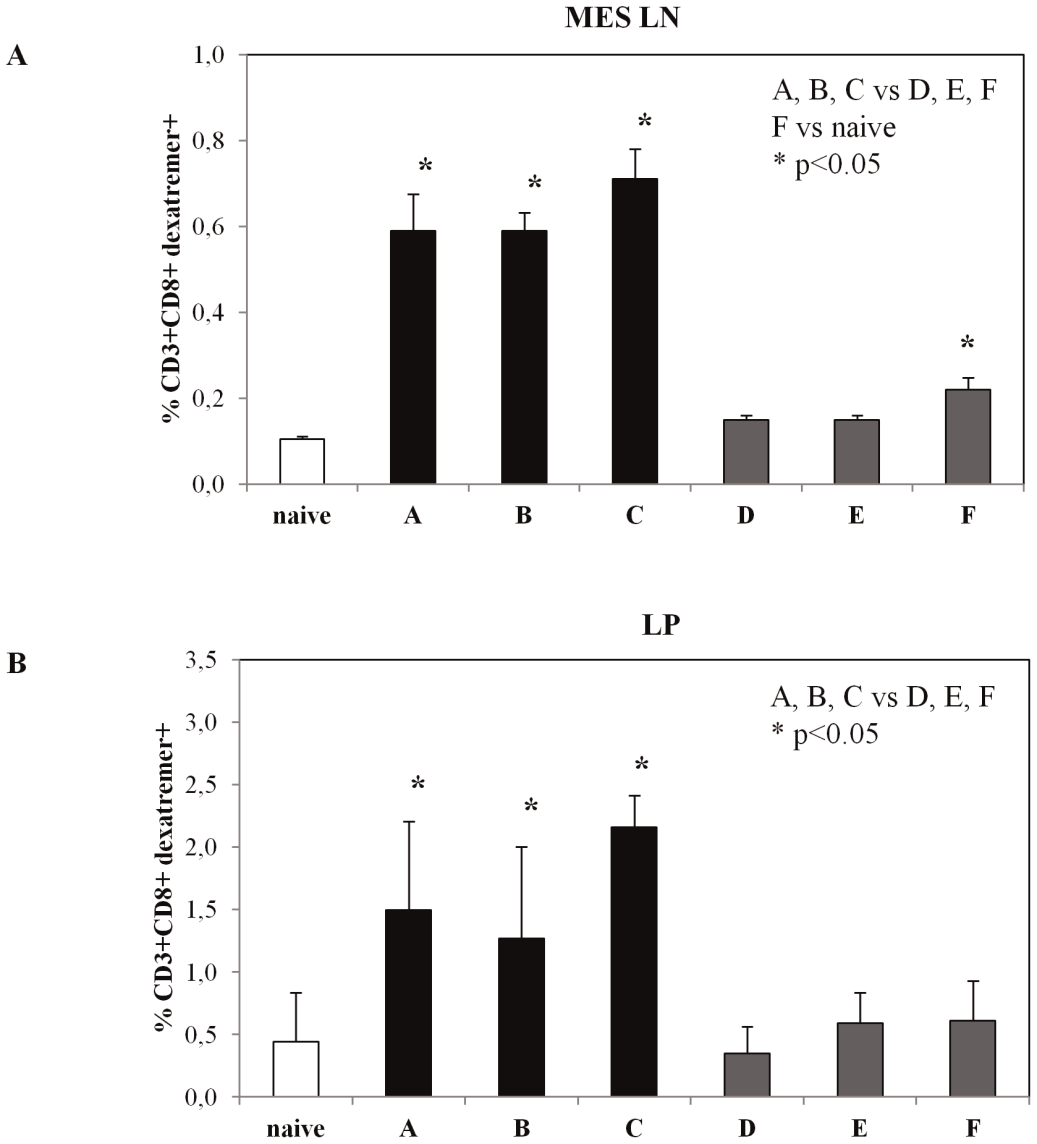
Persistence of mucosal antigen-specific CD8+ T cell response. Six months after the last immunization mice from all groups (vaccination regimens are described in Table 1) were sacrificed. (**A**) Mesenteric lymph node-derived lymphocytes were stained with H-2K^b^-SIINKFEL dextramers, anti-mouse CD3 and anti-mouse CD8. Results are expressed as percentage of CD3+CD8+ dextramers+ cells presented as group means ± standard deviations. The asterisks indicate statistically significant differences (p<0.05) between indicated groups. (**B**) Lymphocytes derived from large intestine *lamina propria* (LP) of mice from indicated group were stained with fluorescent H-2K^b^-SIINKFEL dextramers, anti-mouse CD3 and anti-mouse CD8. The analysis was performed on gated CD3+CD8+ cells from immunized or naïve mice. Results are expressed as percentage of CD3+CD8+ dextramers+ cells presented as group means ± standard deviations. The asterisks indicate statistically significant differences (p<0.05) between indicated groups.

To evaluate the persistence of vaccine-induced CD8+ T cell responses at systemic distal and mucosal sites, we measured the frequency of OVA-specific CD8+ T cells in mesenteric lymph nodes and in LP lymphocytes by dextramer staining (Fig. 6). OVA-specific CD8+ T cells were found in mesenteric lymph nodes from all groups of mice immunized with IDLV-OVA (groups A, B and C) (Fig. 6A), but not in those derived from groups D, E and F. The same analysis was performed on LP derived lymphocytes (Fig. 6B). CD8+ dextramer+ lymphocytes were present in all IDLV-OVA immunized mice (groups A, B and C) but no longer detectable in the other groups that were positive at 2 weeks after immunization (groups E and F, cf. Fig. 3).

**Figure 7:**
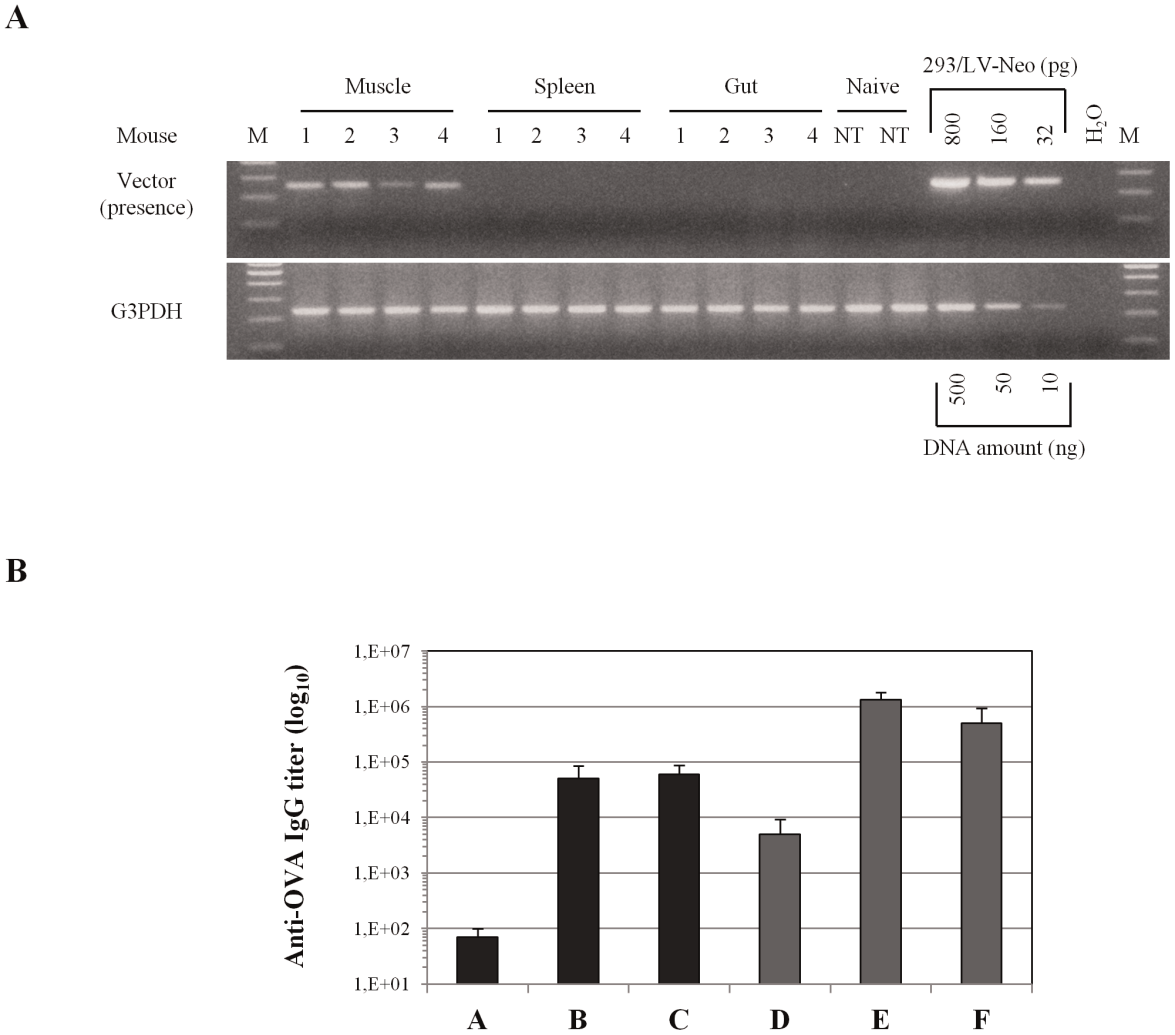
Persistence of IDLV in the immunized mice. (**A**) PCR analysis for evaluation of vector presence in DNA extracted from indicated tissue samples at 6 months from IDLV-OVA injection. The 293 cell line stably transduced with the TY2-GFP-IRES-Neo vector (293/LV-Neo) was used as standard for evaluating vector presence, as already described [54]. DNA quality and integrity of all samples was evaluated by PCR amplification of GAPDH on 200 ng of DNA. PCR samples were run on a 2% agarose gel. G3PDH, glyceraldehyde 3-phosphate dehydrogenase; NT, muscle from naïve mice. **Persistence of anti-OVA IgG in plasma.** (**B**) Anti-OVA IgG titer in plasma samples collected from immunized mice at 6 months after the final immunization (vaccination regimens are described in Table 1). Results are expressed as mean titer presented as group means ± standard deviations.

Given the prolonged immune response observed after IDLV-OVA immunization, persistence of the vector at systemic and mucosal sites was evaluated by DNA-PCR in mice vaccinated with IDLV-OVA at 6 months after the single immunization (group A). As shown in Figure 7A, while the presence of the vector was detected in all muscle samples (injection site), we did not find evidence of lentiviral vector sequences at systemic sites, including spleen and gut (large intestine). No vector sequences were detected in tissue samples from naïve mice. This is in line with previous studies showing persistence of IDLV sequences at the injection site [23], [25].

Finally, the persistence of antibody response was evaluated in plasma and in mucosal secretions. Plasma anti-OVA IgG were still present in all groups of mice at 6 months after the final immunization, although at titers lower than those observed at 2 weeks after the final immunization (Figure 7B). Conversely, no detectable anti-OVA IgA were found in mucosal samples from the immunized mice, regardless of the immunization protocol (data not shown).

## Discussion

Whereas the efficacy of IDLV immunization at inducing prolonged systemic antigen-specific immune responses is now well established, data are still lacking concerning the IDLV-induced mucosal immune responses. We previously demonstrated that a single intramuscular injection of IDLV induced systemic antigen-specific CD8+ T cell responses that were detectable up to 3 months in spleen, draining LN and bone marrow derived cells [23], [25], [39]. Here we showed for the first time that antigen-specific CD8+ T cells induced after a single i.m. immunization with IDLV-OVA were detectable at both systemic and mucosal sites, including distal LN and the *lamina propria* of large intestine, for up to 6 months after the immunization. These results are in agreement with data demonstrating that recombinant adenoviral vectors, an efficient vaccine delivery system, induced mucosal cellular response after intramuscular immunization in mice and non human primates [40].

The cellular immune response induced by IDLV was compared in heterologous prime-boost vaccine strategies, based on the immunization with the same antigen delivered as a soluble protein either i.m. or s.l. together with LT as an adjuvant. It has been extensively demonstrated that LT is a potent adjuvant for vaccines delivered through both systemic and mucosal routes of immunization [41], [42]. Our data clearly demonstrated that i.m. immunization with IDLV induced the highest T cell responses and showed an overall superior efficacy in terms of persistence at mucosal site, where it appears necessary for the maintenance of antigen-specific CD8+ T cells. Interestingly, the s.l. boosts with OVAp + LT did not increase the T cell response induced by IDLV priming, while in the mice s.l. immunized twice with OVAp +LT, the boost with IDLV resulted in a significantly higher T cell response both in the spleen and in draining LN. Since IDLV immunization induced high levels of effector CD8+ T cells [23], we can hypothesize that a boost given during the peak of response could have been detrimental. Further experiments aimed at analyzing this phenomenon are warranted, such as analysis of exhaustion markers on T cells and use of longer intervals between prime and boost(s). On the other hands, the frequency of antigen-specific CD8+ T cells measured several months after the last s.l. boost was still high in spleen, mesenteric LN and in LP of large intestine, and comparable to that observed in the other groups immunized with IDLV-OVA alone or as a boost.

In addition to T cell responses, we also evaluated the antibody response at systemic and mucosal sites. Results clearly indicated that intramuscular injection of IDLV-OVA alone induced low levels of antigen specific antibodies. This is consistent with our previous work using plasmid DNA or IDLV expressing HIV-Env [23], [39] and in line with data showing that DNA or other vector-based immunizations are poor inducers of antibody response [43], [44], especially compared to i.m. immunizations with soluble antigens delivered in combination with strong adjuvants. In fact, our results showed that i.m. immunization with 20 µg of soluble OVA together with the potent adjuvant LT induced higher antibody titers than i.m. immunization with IDLV (Fig. 4). When the same dose of protein and adjuvant was given once through the s.l. route, known to be less efficient than i.m. route, anti-OVA IgG levels in plasma were comparable to those found in mice immunized with IDLV. As expected, priming with IDLV-OVA followed by two s.l. doses of LT-adjuvanted soluble OVAp strongly increased the systemic antibody response, although at levels significantly lower than those observed in mice intramuscularly primed with OVAp + LT and boosted twice mucosally.

However, only by using the mucosal route of immunization we were able to detect anti-OVA IgA in mucosal secretions. Interestingly, in mice primed with IDLV and boosted twice sublingually, mucosal anti-OVA IgA were produced at levels similar to those present in mice immunized sublingually four times, considered our positive control for mucosal responses. Our results also suggest that IDLV as a prime (group B) but not as a boost (group C) was able to drive the mucosal IgA response in combination with mucosal immunizations. Since the vector persists at the site of injection, we can speculate that the persistent antigen expression from IDLV in mice primed with the vector helped in driving a better production of both systemic and mucosal antibodies when boosted with OVAp + LT. We did not evaluate the presence of antibody secreting cells at mucosal sites, therefore we cannot exclude that IgA present in mucosal secretions might originate from blood. Since it has been demonstrated that s.l. immunizations using bacterial toxins as adjuvants in combination with different antigens induced local production of IgA [4], [5], we can only speculate that, also in this study, IgA in mucosal secretions were produced locally.

We also showed that at 6 months after the final immunization anti-OVA IgG antibodies were still present in plasma in all groups of immunized mice, suggesting that all vaccine strategies used in this study efficiently induced long term systemic antibodies. Conversely, mucosal IgA antibodies were transient, and became undetectable in all groups at later time points (data not shown), suggesting that further mucosal boost(s) may be necessary to maintain the mucosal IgA antibody response. Alternatively, immunization schedules with longer intervals may induce higher mucosal and systemic antibody responses, as already demonstrated in different settings [16].

We previously demonstrated that 3 months after a single IDLV immunization the vector was still detectable in muscle samples from the injection site [23], [25]. Here we showed that the vector is detectable at 6 months from the immunization only at the site of injection (Fig. 7B). This is in agreement with reports showing that IDLV persists up to 9 months from the inoculum in non-dividing cells or tissues [45]–[49]. It can be hypothesized that transduction of non-proliferating cell populations at the injection site, including muscle fibers and professional antigen presenting cells, ensures persistence of the vaccine antigen and consequently prolonged presentation to naïve T cells in draining lymph nodes. However, although IDLV-OVA is expression competent and it is an ideal tool for induction of CD8 T cell response, the amount of protein it produces may not be sufficient to induce or boost high levels of antibodies. Development of heterologous prime-boost regimens combining IDLV-OVA with soluble protein + adjuvant could be useful to this aim. Other groups showed that following immunization with lentiviral vectors, the antigen presentation to CD8 T cells persists more than 40 days in an adoptive transfer model [26], [50]. This feature may be responsible for the induction of the long term immune response induced by IDLV. Further characterization of the microenvironment and the mechanism of antigen presentation associated with IDLV injection could help to better clarify this issue. Concerning safety issues, IDLVs are non-replicating and non-integrating lentiviral vectors, produced by incorporating a mutated form of the integrase protein in the recombinant lentiviral particles. Absence of integration has been demonstrated in several murine models *in vivo* and in cell culture model systems [51]. Importantly, a recent report showed that, compared to the parental integrating counterpart, the genotoxicity of IDLV-associated insertional mutagenesis was negligible, making IDLV highly attractive from a biosafety standpoint [52].

In this study we showed that systemic immunization with IDLV can overcome immune compartmentalization and generate potent and durable mucosal cellular immunity. Further analysis of mucosal responses after immunization with IDLV using different mucosal routes should be attempted in order to evaluate the ability of IDLV in inducing local mucosal responses, in the absence of toxicity. In this context, we recently demonstrated that two intranasal, but not intramuscular administrations of IDLV expressing Influenza NP were able to protect mice from challenge with a heterosubtypic Influenza virus [53]. However, mucosal responses were not evaluated.

In conclusion, this is the first report demonstrating that i.m. immunization with IDLV induced antigen-specific CD8+ T cells in *lamina propria* of large intestine, an important immune effector site against mucosal associated pathogens, and that the CD8-specific response lasts for a prolonged period of time from the immunization. The response was superior to s.l. or i.m. immunizations with adjuvanted protein. On the other hand, priming with IDLV-OVA alone induced very low plasma anti-OVA IgG levels and failed to induce IgA in the mucosal fluids analyzed, for which mucosal administration of vaccine antigen was required. Among the prime-boost schedules of immunization used in this study the one that better optimized the IDLV-elicited immune response was the IDLV-OVA intramuscular prime and the adjuvanted OVA mucosal boosts. Indeed it induced a more comprehensive immune response in terms of antigen-specific antibodies and CD8+ T cells at mucosal and systemic levels. Our results are preliminary to further studies focused on more relevant antigens in order to assess a possible application for vaccines against important mucosal pathogens.
